# Analyzing Gensini Score as a Semi-Continuous Outcome

**Published:** 2016-04-13

**Authors:** Homa Kashani, Hojjat Zeraati, Kazem Mohammad, Hamidreza Goodarzynejad, Mahmood Mahmoudi, Saeed Sadeghian, Mohammadali Boroumand

**Affiliations:** 1 *Department of Epidemiology and Biostatistics, School of Public Health, Tehran University of Medical Sciences, Tehran, Iran.*; 2 *Tehran Heart Center, Tehran University of Medical Sciences, Tehran, Iran.*

**Keywords:** *Coronary artery disease*, *Coronary angiography*, *Data interpretation, statistical*

## Abstract

**Background:** Investigators frequently encounter continuous outcomes with plenty of values clumped at zero called semi-continuous outcomes. The Gensini score, one of the most widely used scoring systems for expressing coronary angiographic results, is of this type. The aim of this study was to apply two statistical approaches based on the categorization and original scale of the Gensini score to simultaneously assess the association between covariates and the presence and severity of coronary artery disease (CAD).

**Methods:** We considered the data on 1594 individuals admitted to Tehran Heart Center with CAD symptoms from July 2004 to February 2008. The participants’ baseline demographic and clinical characteristics were collected, and their coronary angiographic results were expressed through the Gensini score. The generalized ordinal threshold and two-part models were applied for the statistical analyses.

**Results: **Totally, 320 (20.1%) individuals had a Gensini score of zero. The results of neither the two-part model nor the generalized ordinal threshold model showed a significant association between Factor V Leiden and the occurrence of CAD. However, based on the two-part model, Factor V Leiden was associated with the severity of CAD, such that the Gensini score increased by moving from a wild genotype to a heterozygote (β = 0.44; 95% CI: 0.20-0.69 in logarithm scale) or a homozygote mutant (β = 0.70; 95% CI: 0.28- 1.12 in logarithm scale). The proportional odds assumption was not met in our data ($\chi^{2}$= 54.26; p value < 0.001); however, a trend toward severe CAD was also observed at each category of the Gensini score using the generalized ordinal threshold model.

**Conclusion:** We conclude that besides loss of information by sorting a semi-continuous outcome, violation from the proportional odds assumption complicates the final decision, especially for clinicians. Therefore, more straightforward models such as the two-part model should receive more attention while analyzing such outcomes.

## Introduction

Investigators frequently confront continuous outcomes, wherein a large number of observations equal to zero. In literature, these types of variables are called “semi-continuous”, “zero-inflated continuous”, and/or “clumped at zero” data.^1^ Examples of semi-continuous outcomes are many in medical, economic, and ecological studies. In cardiovascular medicine, the Gensini score, one of the most widely used scoring systems,^2^ is an example where a zero value indicates no luminal stenosis within the coronary artery tree, representing patients without coronary artery disease (non-CAD group).^3^ This is a complete and useful, but not ideal, scoring system developed by Gensini,^3^ which emphasizes more on the severity of CAD. It takes into account the information about the geographical location and degree of luminal narrowing as well as the cumulative effect of multiple obstructions. The severity of stenosis is indicated by the reduction in lumen diameter, and a nonlinear score is assigned to each lesion based upon it. Then, according to the functional importance of the area of each lesion in the coronary tree, a multiplier is applied. The Gensini score is the sum of the lesion scores. Although the Gensini score provides a quantitative variable, it is rarely used quantitatively in statistical analyses. The reason is that it is a semi-continuous outcome with a relatively large number of observed values clustered at zero that cannot be expressed through a single distribution and the right-skewed non-zero values cannot be transformed to normality. 

A simple and common practice is to recode the Gensini score into a dichotomous variable of having CAD or not or to sort it into an ordinal variable using specific cut points. Nonetheless, such an approach leads to loss of information. Alternatively, the two-part approach uses one equation (generalized linear model usually using probit or logit link function) to model the likelihood of having a non-zero value and a second equation (ordinary linear regression) to model the values greater than zero. Even though the two-part model is conceptually attractive, it was developed in econometrics in the early 1980s.^4^ Other statistical methods on modeling semi-continuous data including Tobit^5^ and Heckman sample selection^6^^-^^8^ were also originally described in econometric literature. The application of these models has scarcely been tested in medical studies. Hence, we placed emphasis more statistically rather than clinically on the Gensini score as a semi-continuous outcome and applied two approaches based on the categorization and use of the Gensini score in its original scale to simultaneously assess the association between covariates and the presence and severity of CAD.

## Methods

In this cross-sectional study, we used the data of a published research on the association between Factor V Leiden with the presence and severity of CAD. The details of the data collection procedure and the participants were previously described.^9^ Briefly, a total of 1594 individuals with symptoms related to CAD who were admitted to Tehran Heart Center (Tehran, Iran) for elective coronary angiography between July 2004 and February 2008 were included. Coronary angiography was performed via the percutaneous femoral approach using standard angiographic techniques, and the severity of CAD was expressed with a well-known Gensini score.^3^ The participants’ baseline demographic and clinical characteristics (including age, sex, body mass index [BMI], smoking status, family history of CAD, diabetes, hypertension, hyperlipidemia, creatinine, history of renal failure, left ventricular ejection fraction [LVEF], and Factor V Leiden) were collected after obtaining written informed consent. Concisely, current smokers were those who smoked any kind of tobacco daily or quitted smoking for < 1 month, and any proven CAD in a parent or sibling (under 55 and 65 years for men and women, respectively) was considered as a positive family history of CAD. The genotype analysis for Factor V Leiden was performed using the polymerase chain reaction-based restriction fragment length polymorphism (PCR-RFLP). The local ethics committee approved the study protocol.

After describing the data, we applied two approaches to analysis: the ordinal threshold model based on the categorization and the two-part model based on the original scale of the Gensini score.

As in ordinal threshold model a semi-continuous outcome is grouped into a number of ordered categories so that the first category contains zero outcomes and cut points will be selected to define the other categories,^10^ we defined tertiles of the Gensini score as cut points and applied the logit link function. The relationship between Factor V Leiden and the Gensini score was assessed in unadjusted and adjusted models. Covariates with p values < 0.2 in the univariable analysis were considered in the adjusted model. Although the cumulative odds ratios (ORs) with 95% confidence intervals (CIs) were presented in the results, we found that the crucial proportional odds (PO) assumption, which investigates whether the relationship between the cumulative probabilities of the ordinal outcome categories and the covariates is the same for each category of the outcome, was not met in our data. Therefore, the generalized ordered logit model was fitted afterward, which provides different estimates at each category of the Gensini score.

Two-part model considers a semi-continuous outcome as a mixture of two parts: “occurrence or binary” and “intensity (severity) or continuous”.^4^ With respect to our data, it means that there are two processes: one governs the occurrence of CAD (non-zero vs. zero Gensini score) and the other one manages the severity of CAD (positive values of the Gensini score) conditional on the occurrence of CAD. The logit link function was used in the occurrence part, and the logarithm of the Gensini score was considered in the severity part to handle the skewness conditional where the non-zero Gensini score was observed. The effect of covariates with p values < 0.2 in the univariable analysis at each part of the model was adjusted in a multivariable model to investigate the relationship between Factor V Leiden and the Gensini score. The effects of the covariates on the occurrence of CAD were reported using OR with 95% CI, and their effects on the severity of CAD were presented through β estimates with 95% CI.

Commands ‘twopm’, ‘ologit’, ‘omodel’, and ‘gologit2’ in STATA software (StataCorp. 2011. Stata Statistical Software: Release 12. College Station, TX: StataCorp LP) were used for the statistical analyses. 

## Results

**Figure 1:**
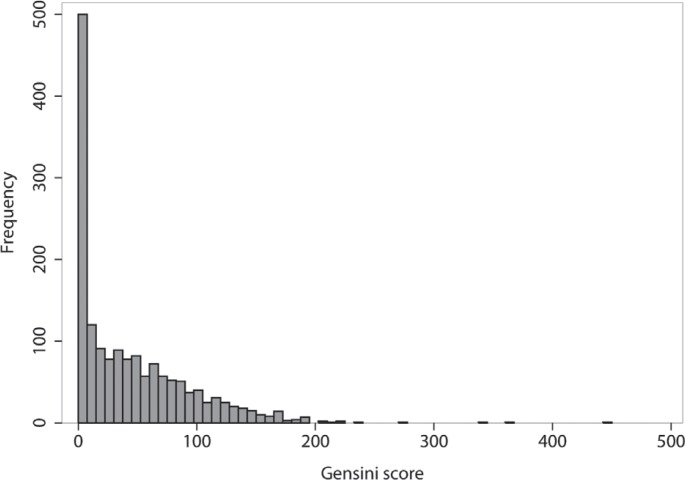
Histogram of the Gensini score of the study participants

The median age of the 1594 participants was 58 years old (1^st^ quartile = 51, and 3^rd^ quartile = 66), and 1022 (64.1%) were male. The individuals’ baseline characteristics are shown in Table 1. The median of the Gensini score was 30.5 (min = 0, and max = 450), and 320 (20.1%) individuals had a zero score (non-CAD group). The histogram of the Gensini score is depicted in Figure 1. The relatively high fraction of zeros and the skewness are obvious in this figure.

**Table 1:** Baseline characteristics of the study participants

	Mean±SD or n (%)
Age (y)	58 (51, 66)*
Sex (male)	1022 (64.1)
BMI (kg/m^2^)	27.77±4.50
Smoking status	
Non-smoker	941 (59.0)
Ex-smoker	306 (19.2)
Current smoker	347 (21.8)
LVEF (%)	52.49±10.74
Diabetes mellitus	449 (28.2)
Hypertension	810 (50.8)
Hyperlipidemia	1044 (65.5)
Creatinine (mg/dL)	1.19±0.50
Family history of CAD	341 (21.4)
History of renal failure	25 (1.6)
Factor V Leiden	
Wild	1456 (91.3)
Heterozygote	106 (6.7)
Homozygote mutant	32 (2)

BMI, Body mass index; LVEF, Left ventricular ejection fraction; CAD, Coronary artery disease

* Median (1^st^ quartile, 3^rd^ quartile)

Ordinal threshold model: The Gensini score cut points for the non-zero values were 24.5 (percentile 33.3) and 68 (percentile 66.7). Therefore, 0, > 0 to ≤ 24.5, > 24.5 to ≤ 68, and > 68 values were considered as Gensini score groups. The univariable cumulative logistic regression models revealed that all the covariates except hypertension (p value = 0.903) had a statistically significant relation with the Gensini score and were considered as potential confounders in the association between Factor V Leiden and the Gensini score. The results of the adjusted ordinal threshold model are shown in Table 2. However, we found that the proportional odds assumption was violated in our data (X^2^= 54.26, df = 22; p value < 0.001). Therefore, the relationship between Factor V Leiden and the Gensini score was not the same in the different classes of the Gensini score and the cumulative OR for the relationship between a heterozygote and a homozygote mutant with the Gensini score relative to a wild genotype could not be considered as the unique estimates of 2.05 and 4.62, respectively.

**Table 2:** Adjusted association between Factor V Leiden and Gensini score using the ordinal threshold model

	OR (95% CI)*	P Value
Factor V Leiden		
Heterozygote	2.05 (1.38-3.05)	< 0.001
Homozygote mutant	4.62 (2.06-10.35)	< 0.001
Age (y)	1.04 (1.03-1.05)	< 0.001
Sex (male)	2.23 (1.75-2.84)	< 0.001
BMI (kg/m^2^)	1.01 (0.98-1.03)	0.625
Smoking status		
Current smoker	1.74 (1.34-2.27)	< 0.001
Ex-smoker	1.37 (1.06-1.79)	0.018
LVEF (%)	0.95 (0.94-0.96)	< 0.001
Diabetes mellitus	1.84 (1.48-2.28)	< 0.001
Hypertension	1.56 (1.24-1.97)	< 0.001
Hyperlipidemia	1.57 (1.28-1.92)	< 0.001
Creatinine (mg/dL)	1.05 (0.82-1.35)	0.698
Family history of CAD	1.56 (1.24-1.97)	< 0.001
History of renal failure	1.41 (0.56-3.57)	0.469

BMI, Body mass index; LVEF, Left ventricular ejection fraction; CAD, Coronary artery disease

* Cumulative OR

The results of fitting the generalized ordered logit model are presented in Table 3. Obviously, due to separate estimations for the cumulative ORs at each category of the Gensini score, it is too complicated to make a general decision for the effect of the covariates. Regarding Factor V Leiden, it was difficult to conclude about the effects; however, the nonsignificant results for Gensini scores > zero versus zero Gensini score might reflect that heterozygote or homozygote mutant genotypes did not have any association with the occurrence of CAD as compared to wild genotype (p value = 0.375 and p value = 0.488) and that they merely played role in the severity of CAD. Despite the complexity of this model, the trend toward a higher Gensini score and, thus, more severe CAD with Factor V Leiden was observed by moving from a wild genotype to a heterozygote (OR = 1.81 and OR = 2.52) or a homozygote mutant (OR = 3.18 and OR = 5.87).

Two-part model: Factor V Leiden was not associated with the occurrence of CAD in the unadjusted model (p value = 0.270). Based on the univariable analysis, we found that the effect of hypertension (p value = 0.230) in the occurrence part and family history of CAD (p value = 0.363) in the severity part needed no adjustment. Table 4 shows the unadjusted and adjusted associations between Factor V Leiden and the occurrence of CAD as well as the severity of disease in the CAD group. Accordingly, Factor V Leiden only had a statistically significant effect on the severity of CAD even after adjustment for the effect of the other covariates (p value < 0.001) and not on its occurrence (OR = 1.25, 95% CI: 0.68-2.31 and OR = 1.42, 95% CI: 0.46-4.43 for heterozygote and homozygote mutant, respectively). In other words, the logarithm of the Gensini score increased by 0.44 and 0.70 by moving from a wild genotype to a heterozygote and a homozygote mutant in the CAD group, respectively.

**Table 3:** Adjusted association between Factor V Leiden and the Gensini score using the generalized ordered logit model

	Gensini score category					
	> 0 vs. 0		> 24.5 vs. ≤ 24.5		> 68 vs. ≤ 68	
	OR (95% CI)*	P Value	OR (95% CI)*	P Value	OR (95% CI)*	P Value
Factor V Leiden						
Heterozygote	1.31 (0.72-2.36)	0.375	1.81 (1.14-2.87)	0.011	2.52 (1.63-3.91)	< 0.001
Homozygote mutant	1.48 (0.49-4.46)	0.488	3.18 (1.29-7.82)	0.012	5.87 (2.58-13.36)	< 0.001
Age (y)	1.06 (1.06-1.07)	< 0.001	1.03 (1.02-1.05)	< 0.001	1.02 (1.01-1.04)	0.001
Sex (male)	2.79 (1.99-3.92)	< 0.001	2.20 (1.67-2.91)	< 0.001	1.69 (1.22-2.33)	0.001
BMI (kg/m^2^)	1.02 (0.99-1.05)	0.220	1.00 (0.97-1.02)	0.718	1.00 (0.97-1.04)	0.795
Smoking status						
Current smoker	1.81 (1.21-2.71)	0.004	2.04 (1.49-2.78)	< 0.001	1.36 (0.97-1.91)	0.071
Ex-smoker	1.57 (1.03, 2.40)	0.035	1.48 (1.09-2.02)	0.013	1.19 (0.85-1.66)	0.316
LVEF (%)	0.95 (0.94-0.97)	< 0.001	0.95 (0.94-0.97)	< 0.001	0.95 (0.94-0.96)	< 0.001
Diabetes mellitus	2.22 (1.57-3.15)	< 0.001	1.69 (1.31-2.18)	< 0.001	1.75 (1.34-2.29)	< 0.001
Hyperlipidemia	1.73 (1.30-2.31)	< 0.001	1.61 (1.27-2.05)	< 0.001	1.38 (1.05-1.81)	0.019
Creatinine (mg/dL)	1.25 (0.76-2.06)	0.386	1.00 (0.75-1.34)	0.990	1.05 (0.80-1.39)	0.722
Family history of CAD	1.92 (1.34-2.75)	< 0.001	1.38 (1.05-1.81)	0.022	1.59 (1.19-2.13)	0.002
History of renal failure	1.21 (0.10-14.71)	0.884	1.39 (0.44-4.39)	0.575	1.63 (0.60-4.42)	0.337

BMI, Body mass index; LVEF, Left ventricular ejection fraction; CAD, Coronary artery disease

* Cumulative OR

**Table 4:** Association between Factor V Leiden and the Gensini score using the two-part model

Model	Variables	Occurrence of CAD		Severity of CAD	
		OR (95% CI)	P Value	β (95% CI)*	P Value
Unadjusted					
Factor V Leiden					
Heterozygote		1.35 (0.79-2.31)	0.267	0.49 (0.24-0.74)	< 0.001
Homozygote mutant		1.81 (0.63-5.20)	0.271	0.89 (0.46-1.33)	< 0.001
Adjusted					
Factor V Leiden					
Heterozygote		1.25 (0.68-2.31)	0.471	0.44 (0.20-0.69)	< 0.001
Homozygote mutant		1.42 (0.46-4.43)	0.543	0.70 (0.28-1.12)	0.001
Age (y)		1.06 (1.04-1.08)	< 0.001	0.01 (0.00-0.02)	0.003
Sex (male)		2.99 (2.12-4.23)	< 0.001	0.28 (0.11-0.45)	0.001
BMI (kg/m^2^)		1.02 (0.99-1.05)	0.202	-0.01 (-0.02-0.01)	0.483
Smoking status					
Current smoker		1.72 (1.14-2.59)	0.010	0.23 (0.05-0.40)	0.010
Ex-smoker		1.51 (0.99-2.32)	0.057	0.12 (-0.05-0.29)	0.157
LVEF (%)		0.96 (0.94-0.97)	< 0.001	-0.02 (-0.03 - -0.02)	< 0.001
Diabetes mellitus		2.27 (1.59-3.24)	< 0.001	0.17 (0.03- 0.31)	0.015
Hypertension		-	-	-0.08 (-0.22-0.05)	0.239
Hyperlipidemia		1.73 (1.29-2.32)	< 0.001	0.27 (0.13-0.41)	< 0.001
Creatinine (mg/dL)		1.31 (0.76-2.25)	0.325	-0.02 (-0.17-0.12)	0.782
Family history of CAD		1.87 (1.30-2.71)	0.001	-	-
History of renal failure		1.41 (0.14- 14.41)	0.774	0.22 (-0.34-0.77)	0.441

BMI, Body mass index; LVEF, Left ventricular ejection fraction; CAD, Coronary artery disease

* For logarithm of the Gensini score

## Discussion

In the present study, the ordinal threshold and two-part models were applied to simultaneously assess the association between Factor V Leiden and the occurrence and severity of CAD using a semi-continuous Gensini score. Using the ordinal threshold model, zero values were considered as one group and the other values were classified. We found that PO assumption was not met in our data, so separate estimates for the aforementioned relationship in the different classes of the Gensini score were presented. Several estimates for the cumulative ORs provided a complicated model, which makes it difficult, especially for clinicians, to come to a clear conclusion. As Min and Agresti^1^ mentioned, arbitrary selection of the cut points and the number of categories as well as loss of information due to categorizing a continuous variable are among other disadvantages of this approach. Using this model, we found it difficult to arrive at a conclusion regarding the association between Factor V Leiden and the severity of CAD since it varies in the different categories of the Gensini score. Nevertheless, a trend from a nonsignificant association with the occurrence of CAD toward a stronger association with the severity of CAD was observed by moving from a wild genotype to a heterozygote or homozygote mutant.

A more appropriate approach, the two-part model with the characteristic of considering two processes for the occurrence and severity of disease was then applied. The comprehension and interpretation of this approach is straightforward, especially for clinicians. The two-part model was preferred to its Tobit or Heckman sample selection counterparts since the Tobit model takes into account the same influence for covariates on the occurrence and severity of CAD.^5^ However, as was observed in our data, this might not always be the case. Also, we avoided using the Heckman sample selection model because zeros in the Gensini score are actual and not missing or censoring data.^11^^, ^^12^ Findings based on this model clearly revealed that Factor V Leiden was not significantly associated with the occurrence of CAD neither before nor after adjustment for the effect of the other covariates. Nevertheless, when CAD happened, this factor was associated with its severity, such that as compared to wild genotype, heterozygote and homozygote mutant were associated with an increase in the Gensini score values, indicating more severe CAD. Although the trend was also shown using the generalized ordinal threshold model, it was based on the estimation of a number of ORs. It was very easily concluded through single β estimate from the two-part model. Another salient point is that the number of estimated ORs depends on the arbitrary selection of the number of cut points. Using these data before and considering the vessel score in analyses, Boroumand et al.^9^ reported the existence of an association between Factor V Leiden and both the occurrence and the severity of CAD; however, using a more informative scoring system, we did not observe this relation for the occurrence of CAD. This can be explained by the difference in the classification of CAD patients. In the current study, the non-CAD group was considered as individuals with a zero Gensini score (normal subjects), whereas Boroumand et al.^9^ considered normal and minimal (< 50% luminal stenosis) individuals as the non-CAD group. Therefore, individuals with a small Gensini score who might be in the minimal classification of the vessel score were considered in the CAD group in the present study. Hence, it appears that one should be cautious about combining minimal coronary with normal subjects.

Chang et al.^13^ concluded that only PO assumption played a role in selecting between the two-part and ordinal threshold models and that only in the case of PO assumption failure was it possible for the predictors of zero values to be different from those of other values; otherwise, there was no priority in choosing between these two approaches. However, in our opinion, the nature of data is very important. In our data, zero values for the Gensini score represent normal individuals without luminal stenosis, and separating them from others seems rational when investigating the effect of covariates or doing adjustment to study some favorable relationship.

In this study, we used logit and logarithm link functions for the two parts of the model because it is easy to interpret the results. The link function for the occurrence part is limited to logit or probit; however, attempts have been made to consider other link functions such as gamma or log-skew-normal for the severity part of the model.^14^^, ^^15^ Another important issue worth noting is that since longitudinal and repeated measurements designs are commonly observed in medical studies, statisticians have recently focused more on providing suitable methods for modeling semi-continuous outcomes in these situations usually by considering random-effects in models.^16^^-^^20^

In this study, we focused on the Gensini score. Be that as it may, interest toward using other scoring systems such as the SYNTAX score^21^ has been raised recently. The Gensini score is a good example of a semi-continuous outcome in clinical studies. A relatively high proportion of zero values and right skewed distribution might be frequently observed using other outcomes or scoring systems, and using proper statistical approaches to the analysis of such data will lead to more precise results.

One of the limitations of this study was that we were not able to do a comparison between the applied models easily using routine measures such as the Akaike information criterion (AIC) or Bayesian information criterion (BIC) because this requires using all data in the two models. In the two-part model, the occurrence part uses all the data and the severity part uses just the non-zero data, which leads to the estimation of the pseudo-likelihood rather than the likelihood, whereas the ordinal threshold model provides the likelihood. Therefore, we avoided this comparison, which might be studied more in further research.

## Conclusion

We conclude that besides loss of information by sorting a semi-continuous outcome, violation from the PO assumption complicates arriving at a clear decision, especially for clinicians. Therefore, paying more attention to more straightforward models such as the two-part model is recommended when analyzing such outcomes.

## Notes:

***This paper should be cited as:*** Kashani H, Zeraati H, Mohammad K, Goodarzynejad HR, Mahmoudi M, Sadeghian S, Boroumand MA. Analyzing Gensini Score as a Semi-Continuous Outcome. J Teh Univ Heart Ctr 2016;11(2):55-61.
